# Biological adaptations for cultural transmission?

**DOI:** 10.1098/rsbl.2022.0439

**Published:** 2022-11-30

**Authors:** Thom Scott-Phillips

**Affiliations:** Institute of Language, Cognition Logic and Information, Ikerbasque, San Sebastian, Spain

**Keywords:** adaptation, culture, cultural evolution, evolutionary psychology, social minds, cognitive development

## Abstract

According to several interlinked and influential lines of argument, human minds have been shaped by natural selection so as to include biological adaptations with the evolved, naturally selected function to facilitate the transmission of cultural knowledge. This ‘cultural minds’ hypothesis has proved highly influential, and if it is correct it is a major step forward in understanding how and why humans have survived and prospered in a hugely diverse range of ecologies. It can be contrasted with a ‘social minds’ hypothesis, according to which cultural transmission occurs as an outcome, but not the biologically evolved function, of social cognition the domain of which is relatively small-group interaction. Here, I critique the cultural minds hypothesis and I argue that the data favour the social minds perspective. Cultural phenomena can clearly emerge and persist over time without cognitive adaptations for cultural transmission. Overtly intentional communication plays an especially pivotal role.

## 1. Introduction

Cultural phenomena—both mental representations (knowledge, beliefs and desires) and their public expressions (words, behaviour and artefacts)—are enormously varied. Some are fleeting and limited to specific groups (in-jokes, gossip and fashions), some are long lasting and can spread to all corners of large populations (supernatural beliefs, children's games and material artefacts), and some have a clear cumulative character (technologies, tools and scientific knowledge). Furthermore, humans have survived and prospered in physical environments as diverse as deserts, forests and polar regions, and this ecological success clearly relies on the transmission and retention of cultural knowledge ([1–6]; *inter alia*). These facts raise fundamental questions about adaptation and ecology in humans. Why are we so heavily cultural? Why are other species less so? What, if not adaptation for culture, explains humans' massive ecological spread?, (and what is culture anyway, from a scientific point of view?: see [7–10]).

One prominent view is that the ordinarily developing human cognitive phenotype includes capacities specialized for the purpose of acquiring, transmitting and organizing culture (table 1). This ‘cultural minds’ hypothesis has proved highly influential, providing a foundation for several research programmes in biology, anthropology, psychology and economics ([5,17–20]; *inter alia*), and even for proposed meta-frameworks for the human sciences [21,22]. The cultural minds hypothesis can be contrasted with a ‘social minds’ perspective, according to which cultural transmission occurs as a consequence of cognitive capacities the proper domain of which is ordinary, one-to-one or one-to-few social interaction. The simple difference here is between *outcome* and *evolved function*. Humans are highly social and this generates recurrent patterns of belief and behaviour at the level of the population: none of this is in any doubt. However, this does not necessarily mean that human minds have been specifically shaped by biological natural selection *in order to* enable cultural transmission ([23, p. 30]; [24, p. 252]; [25]). ‘Predisposed’ and ‘prone to’ are not the same as ‘shaped by natural selection for’ and ‘evolved to’.

**Table 1.** *The ‘cultural minds’ hypothesis*. (Representative statements of the cultural minds hypothesis: that the ordinarily developing human cognitive phenotype includes processes targeted at cultural transmission *qua* cultural transmission. Italics were added in all cases.)

‘brains that have been *shaped by natural selection* to learn and manage culture’	Richerson & Boyd [1, p. 7]
‘cognitive mechanisms that enable the transmission of cultural knowledge… represent *an evolutionary adaptation*’	Csibra & Gergely [11, p. 1149]
‘a psychology *adapted for* cultural learning’	Apicella & Silk [12, p. R450]
‘children have powerful cognitive systems *evolved to* acquire quite complex cultural knowledge from their elders'	Richerson *et al*. [13, p. 7]
‘psychological adaptations that *evolved to* improve the quality of information acquired via cultural transmission’	Henrich & Gil-White [14, p. 165]
‘cognition *specifically adapted* for cultural life’	Laland [5, p. 230]
‘our *genes* have evolved… adaptations that have *wired our minds and bodies for culture*’	Pagel [15, p. 7]
‘a long childhood is *devoted to* acquiring this extensive cultural heritage and may well represent *a human adaptation*’	Whiten & Erdal [16, p. 2122]

Here I summarize two empirically oriented and complementary lines of argument. First, on the more critical side, I examine the cultural minds hypothesis through the lens of cognitive development and organismal design. Then, more forward-looking, I summarize a social minds perspective on culture, highlighting in particular how the flexible and open-ended nature of human communication facilitates the flow of information in human groups.

## 2. Identifying cognitive adaptation

In the development of the cultural minds hypothesis, one main focus has been cumulative phenomena such as technology, and a central idea has been that humans have distinctive cognitive capacities of imitation and accompanying heuristics or learning strategies (prestige bias, conformity bias) that facilitate the acquisition of adaptive information. Much of the research said to support the hypothesis can be grouped into three types: (i) formal models describe the evolutionary conditions in which social learning of cultural information can produce adaptive outcomes ([3,18,26,27]; *inter alia*); (ii) experiments with adults show how they behave in ways similar to those assumed in the modelling work, and more broadly in ways consistent with the cultural minds hypothesis ([28–31]; *inter alia*); and (iii) experiments with human infants have revealed a readiness to copy novel actions that are plausibly indicative of culturally relevant information, even though they appear causally irrelevant, e.g. tapping a box before opening it, even when the tapping makes no difference to whether or how the box is opened ([32–34]; *inter alia*). These bodies of work collectively show how humans are predisposed to acquire cultural items from others, and they help to describe how these predispositions generate and stabilize behaviours, skills and artefacts in human populations.

However, most of this research does not directly target cognitive processes as such, but rather the behavioural outputs of cognition ([24,35,36]; *inter alia*). In some cases, an agnosticism about underlying cognition is an overt strategic choice: ‘The SLS (social learning strategies) literature has been explicit from the outset in disavowing any commitment to mechanism’ [37, p. 657]. In other cases, the behavioural and the cognitive levels of analysis are simply conflated. For instance, reviewing Henrich's book *The secret of our success* [4], which presents an influential version of the cultural minds hypothesis, Clarke & Heyes observe that, ‘cognition—how the mind works—is central to Henrich's analysis, and yet cognitive science is almost completely absent … the interpretive framework is always folk psychological … cognitive science is absent without leave’ [38, p. 292].

How can we identify the effects of biological natural selection in cognition? In general, an evolutionary perspective on organismal design suggests that adaptations should exhibit evidence of design for well-identified, recurrent problems of high-adaptive importance, and should emerge at the adaptively appropriate stage of ontogeny. This agenda is significantly complicated in the human case because of the pervasive influence of cultural scaffolding, which generates a great many cognitive skills that show evidence of design but which cannot be said to be biological adaptations in any straightforward sense [39–41]. The clearest examples are literacy and numeracy, often acquired through explicit schooling, but there are countless other examples such as those involved in religious rituals, sports and scientific inquiry. In all these cases, key cognitive skills meet particular, local goals, yet they would not exist if not for the impact of substantial cultural scaffolds.

Arguably the most productive approach to this challenge is to consider cognitive development, where one of the key aims of inquiry is to separate out core cognition from the cultural scaffolds that build upon it. A signature feature of these core cognitive processes is that they ‘embody’ prior assumptions about the world. For instance, processes of visual perception have built into their operation particular expectations that stimuli perceived as objects are physically cohesive, bonded, rigid and cannot be acted on at a distance, and violations of these expectations cause cognitive surprise [42,43]. Conceptual development builds on these core foundations as people grow [44]. A now large body of research investigates the possible existence of a stock of such core concepts and capacities in a range of domains, such as object and action recognition, physical space, basic quantities, the biological world, trust, fairness, communication and others ([44–49]; *inter alia*). The most compelling cases exhibit convergent evidence of several types, in particular: (i) specification of input conditions, expected outputs and a computational process to link them; (ii) evidence of design i.e. a transparent link between this computational process and a specific recurrent problem of high-adaptive importance; and (iii) strong empirical evidence that the computational process emerges at an appropriate stage of ontogeny, in a largely predictable way and with relative cross-cultural invariance.

Concordant with this ‘core cognition’ agenda, the most cognitively specified version of the cultural minds hypothesis is arguably the thesis of natural pedagogy [11,50]. The main claim is that when infants are addressed with particular ‘cues’—in particular direct eye contact and motherese—they are predisposed, or ‘biased’, to interpret the behaviour as referential and generic, so as to facilitate the transmission of culturally relevant information. These predispositions are, moreover, argued to be biological adaptations that have evolved by natural selection in reaction to ‘the technological challenge [of] growing up and living in societies that employed more and more sophisticated artefacts and longer and longer means-end sequences' ([11, p. 1154]; see also [51]). The main data said to provide evidence for this view come from experiments of type (iii) above.

Yet these data are better explained as a by-product of the dispositions that govern human communication in general. Crucially, the supposedly ‘biased’ interpretations reliably occur only when the target behaviour has been performed in a communicative way [33]. This triggers in audiences the same, spontaneous process of interpretation as other ordinary cases of communication, governed by a presumption of optimal relevance which delivers the (incorrect) conclusion that the demonstrated actions are useful, even if that utility is currently opaque to the audience ([24, p. 244–245]; [52]). In short, the observed data is precisely what is predicted by the social minds hypothesis. Can the cultural minds conclusion still be sustained? Not in the absence of humans' evolved capacities for communication, because then there would be no overtly communicated information to be biased about. Rather, to sustain the cultural minds conclusion, what is necessary is to document how and when the supposed cultural function causes behaviour to deviate from behaviour generated by humans' evolved capacities for communication in general.

In summary: the cultural minds hypothesis is not usually characterized in cognitively precise terms; when it is, there is no compelling evidence of design for the specific purpose of cultural transmission. This conclusion is granted even by researchers who are otherwise sympathetic to the cultural minds hypothesis: ‘clear evidence showing the effect of culture on cognition is lacking’ [25, p. 7915].

However, then how—how just possibly?—can cultural phenomena become so ubiquitous in human groups?

## 3. Culture, without adaptation ‘for’ culture

Multiple, diverse lines of research suggest that many of the most distinctive aspects of human cognition seem to be adaptations to the particular challenge of living in highly interdependent social ecologies. These include, for instance, moral dispositions; an awareness of potential opportunities to exploit others; a strong sensitivity to changes in one's reputation; emotions that help regulate our interactions; distinctive forms of communication, argumentation and deliberation; and several others ([53–62]; *inter alia*). At the same time, this emphasis on social minds cannot fully explain what is distinctive about humans without an accompanying account of how and why human societies are nevertheless so permeated by culture.

There is already, in a different domain of human science, a clear example of how generation and transmission can occur as an outcome, but not the evolved function, of social interaction. That domain is medical epidemiology. Disease is common and widespread in human populations because human bodies are prone to many diseases, even though human bodies have not evolved *in order to* transmit these diseases. There are medical epidemics without there being any biological adaptations the proper function of which is virus transmission. Several researchers have hence suggested how a science of culture could be based on the model of medical epidemiology ([7,63,64]; *inter alia*). Research programmes adopting this epidemiological perspective have helped to explain a wide range of empirical phenomena, such as pseudoscientific beliefs [65] and language change [66], among many others (for reviews see [9,67]). In fact, the word ‘epidemic’ was first coined not to describe something medical but rather something cultural, specifically a particular style of rhetoric and argumentation developed by the Greek philosopher Gorgias in the fourth and fifth centuries BC [68].

Why is culture so much more pervasive in humans than in other species? On this social minds and epidemiological view, the distinctive, overtly intentional character of human communication plays an especially pivotal role [24]. In overtly intentional communication—sometimes called ‘ostensive’ communication—communicators actively reveal their informative intentions to their intended audience [69]. This aligns the interests of communicator and audience. Even if they are otherwise in conflict, such as in a heated argument, individuals engaged in overtly intentional communication share the high-level goal of identification of the communicator's informative intention. This opens up, or ‘unleashes’, the domain of human communication [52], and hence allows humans to communicate about any available topic, to make salient what would otherwise be opaque, to express abstract ideas, to help others learn and more broadly to facilitate the flow of ideas and practices on a grand scale ([70–75]; *inter alia*). Moreover, the mechanistic details of overtly intentional communication help to explain why many otherwise unusual modes of expression are so common in human societies [76–79]. In short, overtly intentional communication is exceptionally dynamic, flexible and open-ended. It is also distinctive of our species ([46,80]; *inter alia*). Thus, coupled with the ordinary observation of others (social learning), overtly intentional communication helps to answer to the question of how and why culture can be so pervasive in humans without any biological adaptations specifically ‘for’ cultural transmission.

Crucially, the cognitive capacities entailed by overtly intentional communication show clear evidence of design for one-to-one and one-to-few interaction, rather than for the spread of information on a larger scale. For instance, if human minds were adapted for larger scale information environments, then when they receive the same information from different sources their epistemic evaluations should be strongly sensitive as to whether or not the sources are truly independent of one another. Yet the data strongly suggest the opposite: humans tend to be not much sensitive to this distinction, while they do tend to perform adaptively for the specific cognitive challenges entailed by small-scale information environments [81,82].

## 4. Conclusion: social minds and epidemiology

The cultural minds hypothesis is not a logical necessity born of the uncontroversial fact that human lives are deeply cultural. It is an empirical claim about evolved cognition in need of independent justification. This justification cannot come simply from the observations that culture is everywhere in human life, that humans readily acquire adaptive information from others, or that cultural items appear similar from one ‘generation’ to the next: because these observations are also all consistent with the social minds hypothesis. What is necessary to show cognitive adaptation is to go beyond surface behaviour to examine the cognitive processes that might be involved ([24,35,83–85]; *inter alia*). There is at present no compelling evidence of cognitive adaptations for the specific tasks of cultural transmission *qua* cultural transmission.

It is possible, of course, that we have not yet investigated in the right way and more supportive data will be later forthcoming. An alternative explanation may be that there are no such dedicated cognitive adaptations, because cultural transmission is not actually a recurrent problem of high-adaptive importance. In ordinary human life, many recurrent problems of high-adaptive importance are social—relationships, fairness, communication and so on—and humans have evolved cognitive capacities to handle all of these. These capacities generate, *in turn*, the flow of information on a scale unmatched in other species.

The cultural minds hypothesis is sometimes advanced almost as a lodestar for the social sciences, but it is cognitively imprecise and its empirical justification is shaky. Viewing human minds as fundamentally social, and culture as epidemiological, are more likely routes towards consilience.

## Data Availability

This article has no additional data.
